# Exploring targets in oropharyngeal cancer – association with immune markers and AI‐scoring of B7‐H3 expression

**DOI:** 10.1002/ctm2.70265

**Published:** 2025-03-12

**Authors:** Jacklyn Liu, Helen Bewicke‐Copley, Shruti Patel, Oscar Emanuel, Nicholas Counsell, Shachi J. Sharma, Volker Schartinger, Oliver Siefer, Ulrike Wieland, Nora Würdemann, Rocio Garcia‐Marin, Jozsef Dudas, Dominic Patel, David Allen, Naomi Guppy, Josep Linares, Adriana Resende‐Alves, David J. Howard, Liam Masterson, Francis M. Vaz, Paul O'Flynn, Cillian T. Forde, Luke Williams, Umar Rehman, John A. Hartley, Johannes Haybaeck, Herbert Riechelmann, Amrita Jay, Tim R. Fenton, Martin D. Forster, Oluyori Adegun, Kerry Chester, Jackie McDermott, Ann Sandison, Manuel Rodriguez Justo, Juan P. Rodrigo, Mario Hermsen, John A. Tadross, Jens P. Klussmann, Matt Lechner

**Affiliations:** ^1^ UCL Division of Surgery and Interventional Sciences London UK; ^2^ UCL Cancer Institute London UK; ^3^ Head and Neck Surgery Cancer Research UK and UCL Cancer Trials Centre London United Kingdom; ^4^ Department of Otorhinolaryngology Head and Neck Surgery University of Giessen Giessen Germany; ^5^ Department of Otorhinolaryngology Head and Neck Surgery University of Cologne Cologne Germany; ^6^ Center for Molecular Medicine Cologne (CMMC) University of Cologne Cologne Germany; ^7^ Department of Otorhinolaryngology Medical University of Innsbruck Innsbruck Austria; ^8^ Institute of Virology, National Reference Center for Papilloma and Polyomaviruses University of Cologne Oviedo Germany; ^9^ Department of Head and Neck Oncology Instituto de Investigacio´n Sanitaria Del Principado de Asturias (ISPA), Instituto Universitario de Oncologı´a Del Principado de Asturias (IUOPA), Centro de Investigacio´n Biome´dica en Red (CIBER‐ONC) Oviedo Spain; ^10^ Department of Histopathology University College London Hospitals NHS Foundation Trust London UK; ^11^ HSL Advanced Diagnostics HSL Advanced Diagnostics London UK; ^12^ Breast Cancer Now Research Centre The Institute of Cancer Research London UK; ^13^ ENT Department Charing Cross Hospital Imperial College Healthcare NHS Trust London UK; ^14^ Royal National Throat, Nose and Ear Hospital and Head and Neck Centre London UK; ^15^ Department of ENT Cambridge University Hospitals NHS Trust Cambridge UK; ^16^ Institute of Pathology Neuropathology and Molecular Pathology Medical University of Innsbruck Innsbruck Austria; ^17^ Diagnostic & Research Center for Molecular Biomedicine Institute of Pathology, Medical University of Graz Graz Austria; ^18^ Faculty of Medicine School of Cancer Sciences Cancer Research UK Centre University of Southampton Southampton UK; ^19^ Department of Histopathology Imperial College Healthcare NHS Trust London UK; ^20^ Department of Histopathology Guys and St. Thomas’ NHS Trust London UK; ^21^ Department of Histopathology and East Midlands & East of England Genomic Laboratory Hub Cambridge University Hospitals NHS Foundation Trust Cambridge UK; ^22^ MRC Metabolic Diseases Unit Wellcome Trust‐Medical Research Council Institute of Metabolic Science University of Cambridge Cambridge UK

1

Dear Editor,

OPSCC has one of the most rapidly increasing incidences of all cancers.^1^, ^2^, ^3^ Most cases are associated with HPV infection, which confers improved survival outcomes, compared to HPV‐independent disease.^4^ Despite advancements in understanding HPV‐associated OPSCC, the role of biomarkers like B7‐H3 and CEA, in both HPV‐positive and HPV‐negative cases remains unclear. This study addresses this gap by evaluating their expression and correlations with clinical and immune parameters. Additionally, we developed a quantitative Digital Image Analysis (DIA) workflow (Supplemental Methods) to assess its feasibility as a companion diagnostic tool for emerging B7‐H3‐targeted therapies.

We observed widespread B7‐H3 expression in OPSCC, while CEA expression was more limited. B7‐H3 is a type I transmembrane protein of the Ig superfamily, which modulates T‐cell activation through receptor binding.^5^ It is overexpressed in cervical cancer (HPV‐driven disease) and Head and Neck Squamous Cell Carcinoma (HNSCC), where it correlates with poor prognosis.^6^, ^7^ We further observed a link between B7‐H3 and CD8+ T‐cell infiltration, which is promising for B7‐H3‐targeted therapies. CEA, an oncofetal protein, is widely used in colorectal cancer but less studied in head and neck cancer. Its established clinical role and widespread use in colorectal cancer support its inclusion in this study for potential clinical translation to OPSCC.^8^

This study included 545 OPSCC cases, with local ethical approval obtained for each institution (Supplemental Methods). The cohort was predominantly male (85.1%) with a mean age of 58.7 years (36–88) (Table 1). Semi‐quantitative assessments and DIA‐based H‐scores were performed to quantify B7‐H3 expression, which was then correlated with clinical outcomes and immune markers. Despite weak staining in most cases, 78.4% exhibited positive B7‐H3 tumoural expression, while 71.0% showed positive stromal expression based on semi‐quantitative assessment, highlighting its widespread prevalence in OPSCC.

**TABLE 1:** Demographic details of the study cohort, where data were available.

	*N* = 545	%
Age (mean, std. dev.)	58.7 (SD: 9.71) (range: 36–88)	
**Gender**		
Male	464	85.1%
Female	81	14.9%
*Total*	545	
**Smoking history**		
None	53	10.6%
Any	448	89.4%
*Total*	501	
**Alcohol history**		
None	128	25.9%
Any	367	74.1%
*Total*	495	
**HPV status**		
HPV‐negative	448	82.2%
HPV‐positive	96	17.4%
*Total*	543	
**T‐stage**		
T1	66	12.2%
T2	165	30.4%
T3	135	24.9%
T4a	148	27.3%
T4b	28	5.2%
*Total*	542	
**N‐stage**		
N0	142	26.3%
≥ N1	397	73.7%
*Total*	539	
**M‐stage**		
M0	508	97.1%
M1	15	2.9%
*Total*	523	
**Grade**		
Well‐differentiated	110	21.4%
Moderately differentiated	244	47.6%
Poorly differentiated	159	31.0%
*Total*	513	

Given B7‐H3's widespread expression in the initial 291 cases, a custom DIA workflow was developed. On these same initial cases, the median tumoural and stromal digital H‐scores were 78.5 (IQR: 27.6–134.6) and 47.4 (IQR: 19.6–81.1), respectively. The semi‐quantitative and H‐scores correlated well for both tumoural and stromal expression (H(3)_Tumour_ = 151.2, *p* < .001; H(3)_Stroma_ = 36.5, *p* < .001; Figure 1). The results were similar when considering HPV‐negative cases only, with strong correlations observed between semi‐quantitative and H‐scores (H(3)_Tumour_ = 119.5, *p* < .001; H(3)_Stroma_ = 30.0, *p* < .001) and a similar pattern of expression as presented above, with median tumoural and stromal H‐scores of 80.3 (IQR: 18.5–157.9) and 54.0 (IQR: 15.0–103.6), respectively. This pipeline was then validated on 235 cases; the median tumoural and stromal H‐scores were 42.8 (IQR: 13.5–109.8) and 31.4 (IQR: 13.0–65.8), respectively.

**FIGURE 1:**
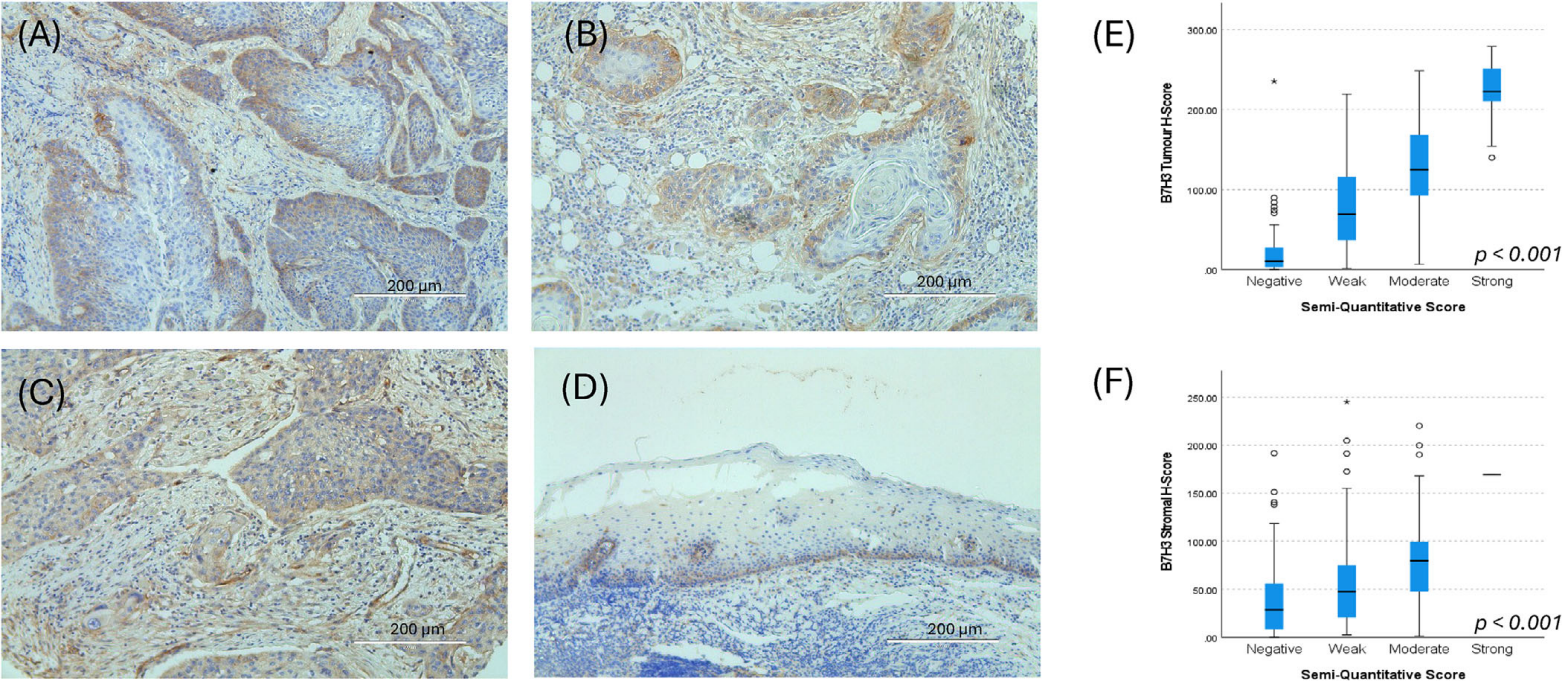
Patterns of B7‐H3 staining observed in oropharyngeal cancer specimens: (A) Representative membranous and cytoplasmic staining, as seen across most cases, where positive staining was observed predominantly in cells at the periphery, adjacent to the stroma and furthest from the centre of tumour nests. (B) Positive staining of a well‐differentiated tumour, where expression is observed in the non‐keratinising cells at the periphery with negative expression in the keratinised interior of the tumour nest. (C) Positive staining of tumour‐associated lymphovascular endothelium. (D) Positive staining in the basal keratinocytes of the normal epithelium. (E, F) Boxplot with medians indicated of B7‐H3 tumoural and stromal H‐score relative to semi‐quantitative grading with statistically significant differences between groups for both the tumour (*p *< .001) and stroma score *(p *< .001).

Associations with clinical factors and prognostic value were evaluated on the overall cohort (*N* = 526). Neither tumoural nor stromal B7‐H3 H‐scores were significantly prognostic (HR_Tumour _= 1.00, 95% CI: 1.00–1.00, *p* = .329; HR_Stroma _= 1.00, 95% CI: 1.00‐1.00, *p* = .556). On univariable analysis, tumoural and stromal B7‐H3 H‐scores significantly correlated with gender, smoking and alcohol history, T‐stage, grade, and HPV‐status (Table S3). When evaluating HPV‐independent cases only, any alcohol history, M0‐stage and lower tumour grade (i.e., more well‐differentiated tumours) were each associated with higher B7‐H3 tumour expression. Higher stromal B7‐H3 scores were also observed with lower tumour grade (Table S4). Lastly, in the subgroup, which was evaluated for immune marker expression, B7‐H3 expression did not significantly correlate with tumoural PDL1 nor PD1 expression. However, reduced CD8+ T‐cell infiltration (*p* = .008) was associated with a higher B7‐H3 tumoural H‐score (Table 2). This significance remained when evaluating HPV‐negative cases only (Table 3).

**TABLE 2:** Cross‐tabulation of B7‐H3 H‐scores in relation to immune marker expression.

		Tumour		Stroma	
	*N* = 29	Mean (std. dev.)	*p*‐value	Mean (std. dev.)	*p*‐value
**PD1**					
Negative	9	82.1 (74.72)	.317	51.9 (45.97)	.660
Positive	20	52.4 (59.36)		38.9 (34.91)	
**PDL1**					
Negative	21	74.6 (70.56)	.324	47.8 (40.40)	.237
Positive	8	27.5 (25.80)		30.1 (30.88)	
**CD8**					
Negative/ poorly infiltrating	13	95.6 (56.87)	***.008***	56.9 (37.66)	***.032***
Moderately/strongly infiltrating	16	34.0 (54.80)		31.6 (36.06)	
**FoxP3**					
Negative	20	75.4 (70.17)	.069	47.5 (41.68)	.444
Positive	9	30.8 (37.40)		42.9 (38.32)	

**TABLE 3:** Cross‐tabulation of B7‐H3 H‐scores in relation to immune marker expression for HPV‐independent cases only.

		Tumour		Stroma	
	*N* = 18	Mean (Std. Dev.)	*p*‐value	Mean (Std. Dev.)	*p*‐value
**PD1**					
Negative	8	92.2 (72.95)	.424	57.3 (46.00)	.477
Positive	10	65.9 (52.54)		38.8 (32.07)	
**PDL1**					
Negative	15	83.7 (65.20)	.594	50.0 (39.49)	.441
Positive	3	47.4 (34.46)		32.0 (38.37)	
**CD8**					
Negative/ poorly infiltrating	11	106.6 (58.11)	***.013***	62.4 (37.71)	***.016***
Moderately/strongly infiltrating	7	32.1 (35.63)		22.9 (27.99)	
**FoxP3**					
Negative	13	88.2 (66.42)	.349	48.1 (41.72)	.961
Positive	5	50.1 (41.79)		44.1 (34.09)	

Two hundred eighty‐five cases were evaluated semi‐quantitatively for CEA expression as a comparator biomarker (Figure S4), 68.8% of which were negative by IHC evaluation. 49.6% of cases demonstrated positive expression of CEA in tumour cells. Several cut‐off values (1%, 5%, 10% and 50%) were explored to evaluate associations between CEA across the tissue section and other clinical factors (i.e., gender, T‐stage, N‐stage, M‐stage, smoking/alcohol history, HPV status and overall survival). No significant correlations were observed except between CEA expression and HPV‐status, which were significantly associated at all cut‐off values, as well as between CEA expression and gender at the 50% cut‐off value (Table S5). On univariable analysis, CEA at each cut‐off was significantly predictive of overall survival, with positive expression correlating with superior survival. However, this significance was lost after adjusting for HPV status (Table S6). In cases for which data were available, CEA did not significantly correlate with any of PD‐1, PDL1, CD8+ T‐cell infiltration nor FoxP3 expression (Tables S7 and S8).

Here, we present clinical findings from a large multi‐centre OPSCC cohort, confirming known prognostic factors. Our DIA workflow enabled high‐throughput B7‐H3 analysis, demonstrating its utility as a companion diagnostic tool given its widespread expression. Notably, HPV‐associated T4 and HPV‐independent T1 cases had similar outcomes, underscoring the aggressiveness of early‐stage HPV‐independent disease and the need for improved therapies, such as those targeting B7‐H3. However, high intratumour heterogeneity in HPV‐independent OPSCC remains a key challenge in developing effective treatments. Interestingly, B7‐H3 correlated with CD8+ T‐cell presence and HPV status, with higher B7‐H3 linked to reduced CD8+ infiltration, even in HPV‐independent cases. CEA was present in a subset of cases, warranting further validation as a therapeutic target in head and neck cancer.

In addition, we observed a more inflamed tumour microenvironment with greater T‐cell infiltration for HPV‐associated disease, compared to HPV‐negative disease, consistent with previous research.^9^ Our findings suggest B7‐H3 plays an immunosuppressive role in OPSCC, which has been shown in a separate study of HNSCC, where the authors further demonstrated an association between B7‐H3 expression and poorer prognosis, highlighting the need for innovative immunotherapies.^10^ Further research should examine B7‐H3's relationship with key OPSCC biomarkers, including PD‐L1, p16, EGFR, and TP53, which have diagnostic and prognostic significance.^1^, ^4^

In conclusion, B7‐H3 plays a key role in both HPV‐associated and HPV‐independent OPSCC and we have demonstrated the feasibility of digital IHC assessment as a potential companion diagnostic tool. Further research is needed to clarify underlying mechanisms and advance immunotherapeutic strategies for OPSCC.

## AUTHOR CONTRIBUTIONS

JL, HBC, SP, OE, NC, SJS, ML, JPK, JAT, MH conducted the data analysis, involved in study design and conception, manuscript preparation and editing. VS, OS, UW, NW, RCM, JD, DP, DA, NG, JL, ARA, DJH, LM, FMV, POF, CTF, LW, UR, JAH, JH, HR, AJ, TRF, MDF, OA, KC, JM, AS, MRJ, JPR, prepared and reviewed the final manuscript.

## CONFLICT OF INTEREST STATEMENT

The authors declare no conflicts of interest.

## FUNDING INFORMATION

The authors received no specific funding for this work.

## ETHICAL STATEMENT

Local ethical approval was obtained from each institution: UCL/UCLH (UCL/UCLH Ethics Committee, 04/0099), HUCA (Ethical Committee of HUCA, 141/19), UHG (Ethics Committee of Giessen, AZ 95/15) and MUI (AN2014‐0241, 340/4.2); multi‐institutional analysis was performed in line with multicenter ethics obtained from UCL (UCL REC no. 9609/002).

## Supporting information



Supporting Information

Supporting Information

Supporting Information

## Data Availability

Data can be provided upon reasonable request by contacting the corresponding author.
