# Supplementary material for: Exploring targets in oropharyngeal cancer – association with immune markers and AI‐scoring of B7‐H3 expression
Source: Clin Transl Med. 2025 Mar 12;15(3):e70265. doi: 10.1002/ctm2.70265 (PMC11897723; doi:10.1002/ctm2.70265)
Supplement: Supplementary file 1 — Supporting Information [file CTM2-15-e70265-s002.docx]

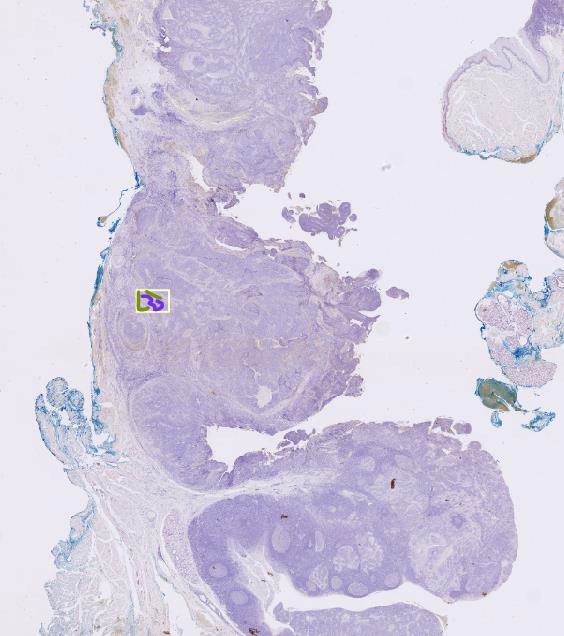

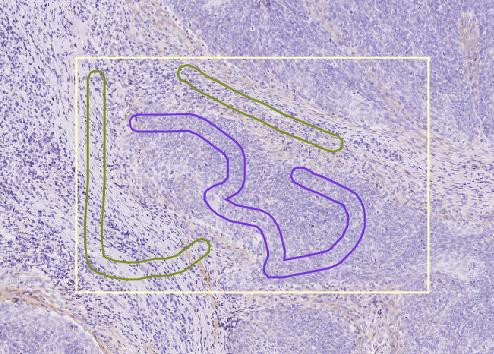

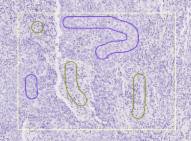

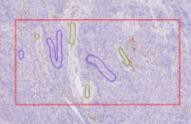

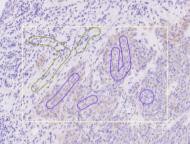

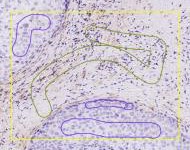

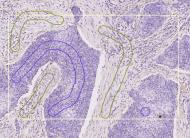

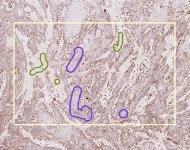

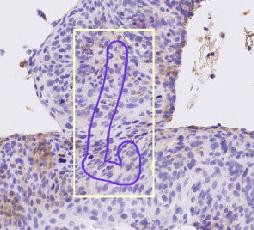

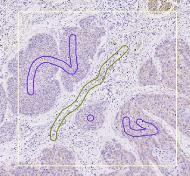

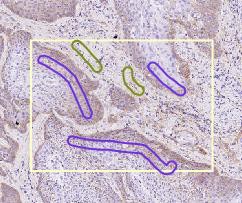

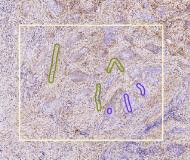

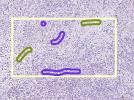

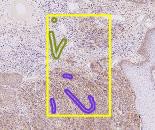

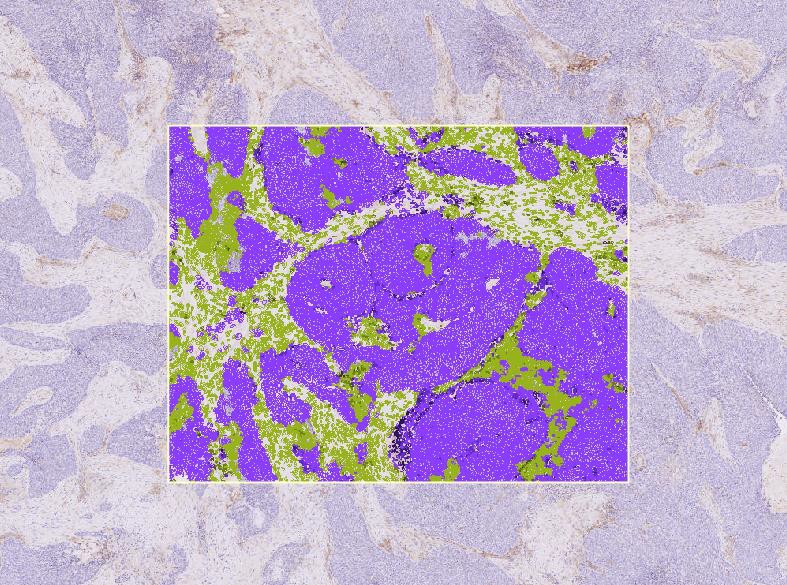


6. Correlation with semi-

quantitative scores (N=291)

1. Scan of B7-H3-stained whole slide (or TMA)

2. Representative regions selected and annotated as tumour (blue) or

stroma (green)

3. Training of tissue classifier on annotated tumour and

stromal regions

**Supplemental Figure 1.** Workflow of the development of an automated digital pathology pipeline for the high through-put evaluation of B7-H3 staining across five hundred twenty-six samples.

4. Classification of individual cells as tumour (blue) or stroma (green)

5. Quantification of positive staining in tumour and

stroma: H-Score

7. Validation on additional cases (N=235) and

integration with clinical data and expression of immune and other markers


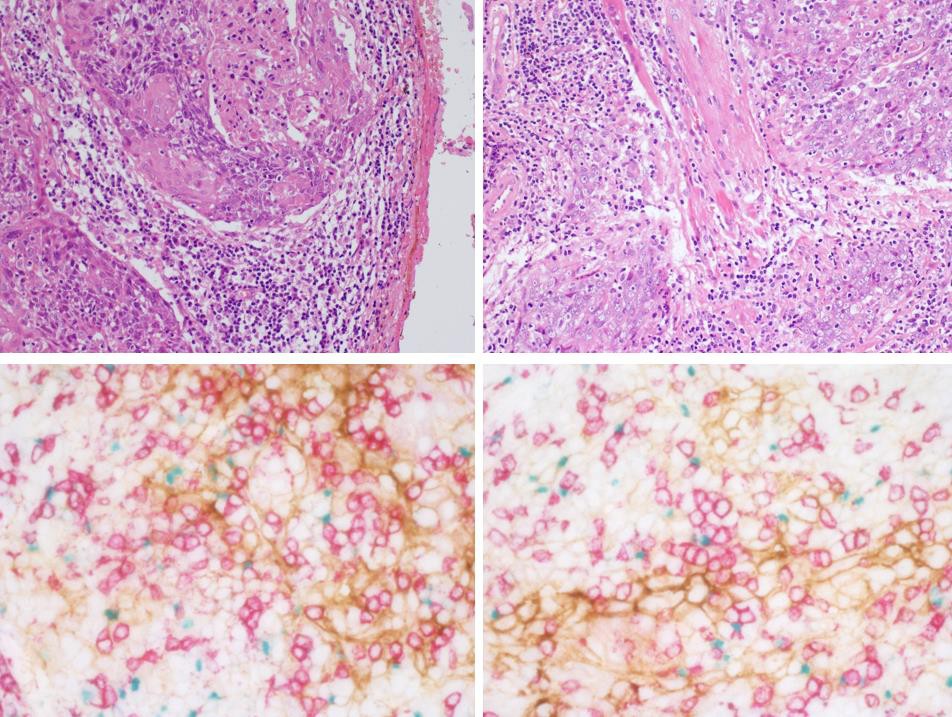

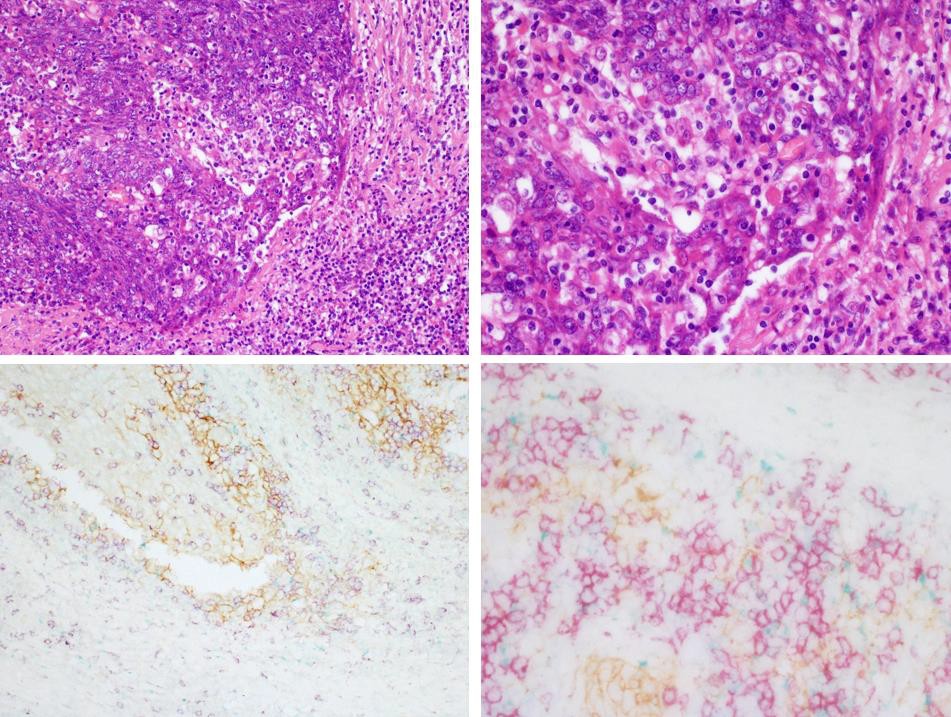
A) B)

*Supplemental Figure 2.* A) HPV-positive SCC: H&E showing moderately differentiated SCC (top). Multiplex IHC shows high expression of PD-L1 in the tumour cells (brown), with rich accompanying population of CD8+ T-cells (red) and increase in T-regs (green) (bottom). B) HPV-negative SCC: H&E showing moderately differentiated SCC (top). Multiplex ICC shows very low expression of PD-L1 in the tumour cells (brown), with tumour associated lymphocytes CD8+ T-cells (red) and occasional T-regs (green) (bottom).


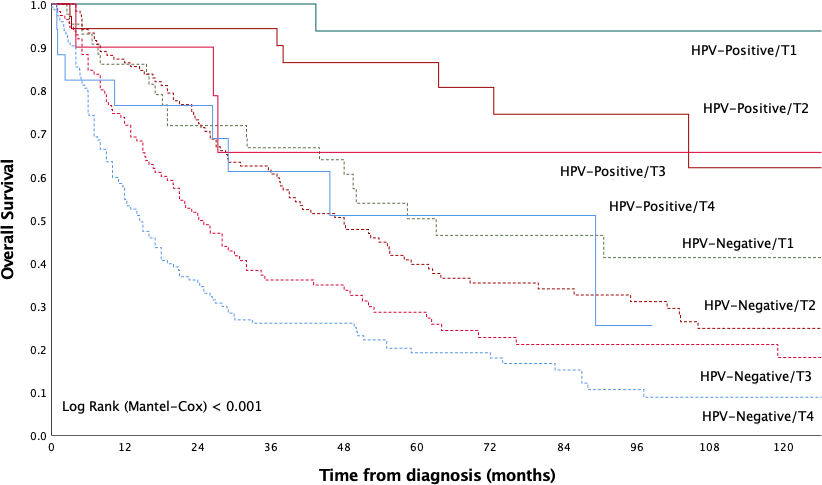


| Number at Risk |  |  |  |  |  |  |  |  |  |  |  |
| --- | --- | --- | --- | --- | --- | --- | --- | --- | --- | --- | --- |
| HPV-Positive/T1 | 17 | 17 | 17 | 17 | 16 | 12 | 9 | 7 | 5 | 2 | 2 |
| HPV-Positive/T2 | 34 | 32 | 29 | 24 | 20 | 17 | 14 | 9 | 7 | 4 | 2 |
| HPV-Positive/T3 | 9 | 9 | 9 | 5 | 2 | 2 | 2 | 1 | 1 | 1 | 1 |
| HPV-Positive/T4 | 16 | 12 | 10 | 7 | 5 | 2 | 2 | 2 | 1 | 0 | 0 |
| HPV-Negative/T1 | 42 | 37 | 30 | 26 | 18 | 14 | 11 | 10 | 6 | 6 | 5 |
| HPV-Negative/T2 | 120 | 99 | 82 | 66 | 52 | 38 | 30 | 24 | 20 | 16 | 15 |
| HPV-Negative/T3 | 115 | 79 | 52 | 32 | 28 | 21 | 14 | 12 | 9 | 8 | 5 |
| HPV-Negative/T4 | 146 | 74 | 46 | 32 | 27 | 17 | 14 | 10 | 6 | 3 | 3 |

**Supplemental Figure 3.** Kaplan-Meier survival curve of T-stage stratified by HPV-status. . Both T-stage and HPV-status were significantly prognostic and, strikingly, on stratification of the former by the latter, a similar overall survival outcome was observed between HPV-positive T4 cases and HPV-negative T1 disease.


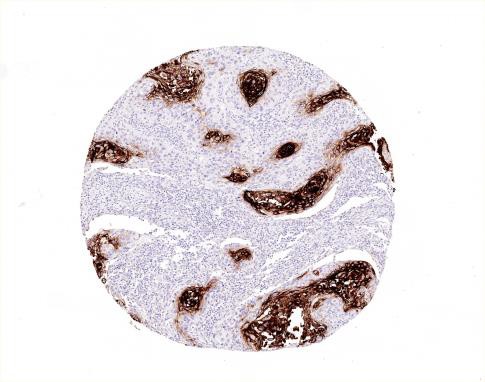

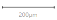

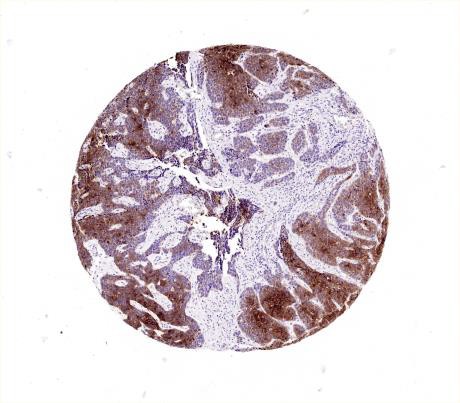

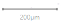

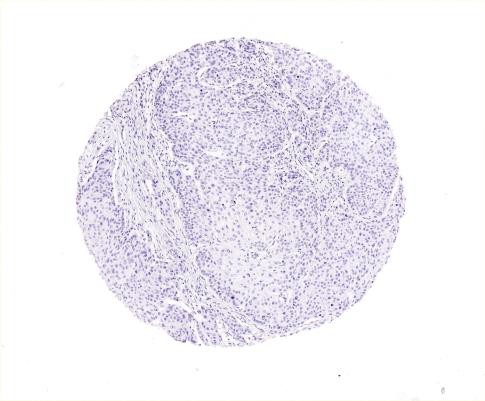

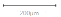


A)

B)

C)

**Supplemental Figure 4: Patterns of CEA staining observed in oropharyngeal cancer specimens in representative TMA cores** (A) strong membrane and cytoplasmic staining in a large proportion of tumour cells; (B) strong cytoplasmic staining in the majority of tumour cells (C) negative staining.
