# Supplementary material for: Exploring targets in oropharyngeal cancer – association with immune markers and AI‐scoring of B7‐H3 expression
Source: Clin Transl Med. 2025 Mar 12;15(3):e70265. doi: 10.1002/ctm2.70265 (PMC11897723; doi:10.1002/ctm2.70265)
Supplement: Supplementary file 2 — Supporting Information [file CTM2-15-e70265-s003.docx]

**Supplemental Tables**

***Supplemental Table 1.*** Univariable and multivariable overall and disease-free survival evaluating the prognostic value of various clinical factors.

|  | **Univariable DFS**  **N=545** | **p-value** | **Multivariable DFS**  **N=545** | **p-value** | **Univariable OS**  **N=545** | **p-value** | **Multivariable OS**  **N=545** | **p-value** |
| --- | --- | --- | --- | --- | --- | --- | --- | --- |
| **Age** | HR = 1.01  (95% CI: 0.99-1.02) | *p*=0.397 | NA | NA | HR = 1.02  (95% CI: 1.01-1.04) | ***p<0.001*** | HR = 1.03  (95% CI: 1.02-1.04) | ***p<0.001*** |
| **Female vs. Male** | HR = 0.38  (95% CI: 0.24-0.60) | ***p<0.001*** | HR = 0.74  (95% CI: 0.44-1.23) | p=0.222 | HR = 0.54  (95% CI: 0.38-0.76) | ***p<0.001*** | HR = 0.96  (95% CI: 0.61-1.52) | p=0.870 |
| **Alcohol History:**  **Any vs. None** | HR = 3.45  (95% CI: 2.42-4.93) | ***p<0.001*** | HR = 2.58  (95% CI: 1.70-3.90) | ***p<0.001*** | HR=2.35  (95% CI: 1.75-3.15) | ***p<0.001*** | HR = 0.21  (95% CI: 1.46-3.01) | ***p<0.001*** |
| **Smoking History:**  **Any vs. None** | HR = 2.02  (95% CI: 1.27-3.22) | ***p=0.001*** | HR = 0.71  (95% CI: 0.42-1.22) | p=0.232 | HR = 1.77  (95% CI: 1.17-2.69) | ***p=0.004*** | HR = 0.81  (95% CI: 0.49-1.34) | p=0.412 |
| **HPV Status:**  **Positive vs. Negative** | HR = 0.30  (95% CI: 0.20-0.43) | ***p<0.001*** | HR = 0.32  (95% CI: 0.17-0.60) | ***p<0.001*** | HR = 0.22  (95% CI: 0.13-0.36) | ***p<0.001*** | HR = 0.30  (95% CI: 0.16-0.54) | ***p<0.001*** |
| **T-Stage** |  | ***p<0.001*** |  | ***p=0.001*** |  | ***p<0.001*** |  | ***p<0.001*** |
| **T2 vs. T1** | HR = 1.48  (95% CI: 0.94-2.33) | NA | HR = 1.03  (95% CI: 0.63-1.70) | NA | HR = 1.66  (95% CI: 1.07-2.59) | NA | HR = 1.39  (95% CI: 0.82-2.33) | NA |
| **T3 vs. T1** | HR = 2.11  (95% CI: 1.33-3.34) | NA | HR = 1.43  (95% CI: 0.86-2.38) | NA | HR=2.73  (95% CI: 1.76-4.26) | NA | HR = 2.25  (95% CI: 1.32-3.82) | NA |
| **T4 vs. T1** | HR = 2.74  (95% CI: 1.75-4.29) | NA | HR = 1.83  (95% CI: 1.12-3.00) | NA | HR = 3.97  (95% CI: 2.58-6.09) | NA | HR = 3.18  (95% CI: 1.89-5.35) | NA |
| **N-Stage:**  **N>0 vs. N0** | HR = 1.24  (95% CI: 0.95-1.61) | p=0.110 | NA | NA | HR = 1.40  (95% CI: 1.09-1.79) | ***p=0.007*** | HR = 1.31  (95% CI: 0.99-1.73) | p=0.057 |
| **M-Stage:**  **M1 vs. M0** | HR = 3.17  (95% CI: 1.68-5.98) | ***p=0.002*** | HR = 4.90  (95% CI: 2.45-9.80) | ***p<0.001*** | HR=2.10  (95% CI: 1.15-3.83) | ***p=0.031*** | HR = 1.91  (95% CI: 0.99-3.67) | p=0.075 |
| **Grade** |  | ***p=0.001*** |  | *p=0.381* |  | *p=0.056* |  | *p=0.834* |
| **G2 vs. G1** | HR = 0.67  (95% CI: 0.50-0.88) | NA | HR = 0.82  (95% CI: 0.61-1.09) | NA | HR = 0.86  (95% CI: 0.66-1.13) | NA | HR = 0.99  (95% CI: 0.74-1.32) | NA |
| **G3 vs. G1** | HR = 0.54  (95% CI: 0.39-0.74) | NA | HR = 0.92  (95% CI: 0.66-1.29) | NA | HR = 0.69  (95% CI: 0.51-0.94) | NA | HR = 1.08  (95% CI: 0.77-1.51) | NA |

***Supplemental Table 2a.*** Expression of immune markers in the initial cohort available for evaluation.

|  | | **n** | **%** |
| --- | --- | --- | --- |
| **PD1** | Negative | 10 | 29.4% |
|  | Positive | 24 | 70.6% |
| **PDL1** | Negative | 26 | 76.5% |
|  | Positive | 8 | 23.5% |
| **CD8** | Negative | 1 | 2.9% |
|  | Poorly Infiltrating | 13 | 38.2% |
|  | Moderately Infiltrating | 14 | 41.2% |
|  | Strongly Infiltrating | 6 | 17.6% |
| **FoxP3** | Negative | 22 | 64.7% |
|  | Positive | 12 | 35.3% |
|  | **Total** | **34** |  |

***Supplemental Table 2b.*** Univariable Cox regression overall survival analysis for the immune markers in the initial cohort available for evaluation.

| **N=34** | | **Univariable OS** | **p-value** |
| --- | --- | --- | --- |
| **PD1** | Positive vs. Negative | HR = 0.68  (95% CI: 0.29-1.62) | p = 0.395 |
| **PDL1** | Positive vs. Negative | HR = 1.33  (95% CI: 0.53-3.33) | p = 0.551 |
| **CD8** | Moderately/Strongly Infiltrating vs. Negative/Poorly Infiltrating | HR = 0.41  (95% CI: 0.18-0.95) | ***p = 0.040*** |
| **FoxP3** | Positive vs. Negative | HR = 0.34  (95% CI: 0.11-0.99) | ***p = 0.028*** |

***Supplemental Table 2c.*** Immune marker expression in relation to HPV-status.

|  | | **HPV-status** | | | | ***p*-value** |
| --- | --- | --- | --- | --- | --- | --- |
|  |  | **Negative** | | **Positive** | |  |
|  |  | **n** | **%** | **n** | **%** |  |
| **PD1** | Negative | 9 | 47.4% | 1 | 6.7% | ***0.020*** |
|  | Positive | 10 | 52.6% | 14 | 93.3% |  |
|  | Total | 19 |  | 15 |  |  |
| **PDL1** | Negative | 16 | 84.2% | 10 | 66.7% | 0.417 |
|  | Positive | 3 | 15.8% | 5 | 33.3% |  |
|  | Total | 19 |  | 15 |  |  |
| **CD8** | Negative | 0 | 0.0% | 1 | 6.7% | ***0.001*** |
|  | Poorly Infiltrating | 12 | 63.2% | 1 | 6.7% |  |
|  | Moderately Infiltrating | 7 | 36.8% | 7 | 46.7% |  |
|  | Strongly Infiltrating | 0 | 0.0% | 6 | 40.0% |  |
|  | Total | 19 |  | 15 |  |  |
| **FoxP3** | Negative | 13 | 68.4% | 9 | 60.0% | 0.724 |
|  | Positive | 6 | 31.6% | 6 | 40.0% |  |
|  | Total | 19 |  | 15 |  |  |

***Supplemental Table 3.*** Cross-tabulation of B7-H3 H-scores in relation to clinical factors, HPV status and immune marker expression.

|  | | | **Tumor** | | **Stroma** | |
| --- | --- | --- | --- | --- | --- | --- |
|  | | **n** | **Mean (Std. Dev.)** | ***p*-value** | **Mean (Std. Dev.)** | ***p*-value** |
| **Gender** | Male | 450 | 80.8 (69.82) | ***0.038*** | 53.6 (46.71) | ***0.019*** |
|  | Female | 76 | 66.5 (69.78) |  | 41.4 (41.30) |  |
| **Smoking** | None | 51 | 48.1 (60.11) | ***< 0.001*** | 39.2 (38.77) | ***0.028*** |
|  | Any | 432 | 83.4 (70.26) |  | 53.8 (46.97) |  |
| **Alcohol** | None | 120 | 53.4 (62.68) | ***< 0.001*** | 41.4 (45.23) | ***< 0.001*** |
|  | Any | 357 | 89.7 (70.64) |  | 56.4 (46.51) |  |
| **T-stage** | T1 | 62 | 55.9 (62.86) | ***0.019*** | 38.6 (41.38) | ***0.013*** |
|  | T2 | 158 | 80.6 (71.88) |  | 50.2 (45.96) |  |
|  | T3 | 130 | 80.0 (70.29) |  | 50.6 (41.38) |  |
|  | T4a/b | 173 | 84.2 (68.89) |  | 59.1 (50.09) |  |
| **N-stage** | N0 | 135 | 82.2 (65.51) | 0.194 | 49.1 (40.77) | 0.883 |
|  | ≥ N1 | 385 | 77.4 (71.18) |  | 52.7 (47.72) |  |
| **M-stage** | M0 | 489 | 80.9 (70.42) | ***0.068*** | 52.5 (46.34) | 0.752 |
|  | M1 | 15 | 47.7 (52.19) |  | 46.3 (39.64) |  |
| **Grade** | Well-diff. | 108 | 91.5 (61.89) | ***< 0.001*** | 56.2 (38.95) | ***< 0.001*** |
|  | Moderately diff. | 235 | 85.7 (74.67) |  | 58.7 (51.70) |  |
|  | Poorly diff. | 152 | 60.3 (62.15) |  | 39.9 (37.51) |  |
| **HPV** | Negative | 413 | 87.5 (71.28) | ***< 0.001*** | 56.3 (47.98) | ***< 0.001*** |
|  | Positive | 78 | 40.8 (48.76) |  | 32.8 (31.66) |  |

***Supplemental Table 4.*** Cross-tabulation of B7-H3 H-scores in relation to clinical factors and immune marker expression in HPV-independent cases, only.

|  | | | **Tumor** | | **Stroma** | |
| --- | --- | --- | --- | --- | --- | --- |
|  | | **n** | **Mean (Std. Dev.)** | ***p*-value** | **Mean (Std. Dev.)** | ***p*-value** |
| **Gender** | Male | 386 | 87.9 (70.81) | 0.355 | 57.0 (47.93) | 0.105 |
|  | Female | 46 | 79.0 (71.62) |  | 46.0 (44.19) |  |
| **Smoking** | None | 26 | 70.9 (66.23) | 0.202 | 52.0 (46.11) | 0.651 |
|  | Any | 379 | 88.3 (71.34) |  | 56.6 (48.03) |  |
| **Alcohol** | None | 69 | 72.1 (68.62) | 0.029 | 53.7 (51.96) | 0.279 |
|  | Any | 335 | 91.4 (71.48) |  | 57.3 (47.19) |  |
| **T-stage** | T1 | 44 | 68.5 (66.28) | 0.194 | 43.9 (45.49) | 0.154 |
|  | T2 | 126 | 93.5 (71.95) |  | 55.8 (48.18) |  |
|  | T3 | 122 | 84.3 (70.91) |  | 52.6 (42.24) |  |
|  | T4a/b | 155 | 87.8 (70.60) |  | 61.0 (51.08) |  |
| **N-stage** | N0 | 122 | 85.8 (63.97) | 0.637 | 50.9 (40.38) | 0.566 |
|  | ≥ N1 | 307 | 86.9 (73.16) |  | 57.3 (49.90) |  |
| **M-stage** | M0 | 407 | 88.6 (71.13) | ***0.033*** | 56.4 (47.72) | 0.479 |
|  | M1 | 14 | 49.9 (53.44) |  | 46.4 (41.13) |  |
| **Grade** | Well-diff. | 102 | 93.0 (60.63) | ***0.013*** | 57.2 (38.21) | ***0.004*** |
|  | Moderately diff. | 209 | 92.6 (76.60) |  | 62.3 (53.56) |  |
|  | Poorly diff. | 111 | 70.8 (64.30) |  | 43.3 (39.36) |  |

|  | | **1 % p-value** | | | **5%**  **p-value** | | | **10%**  **p-value** | | | **50%**  **p-value** | | |
| --- | --- | --- | --- | --- | --- | --- | --- | --- | --- | --- | --- | --- | --- |
|  |  | **Negative**  **n (%)** | **Positive**  **n (%)** |  | **Negative**  **n (%)** | **Positive**  **n (%)** |  | **Negative**  **n (%)** | **Positive**  **n (%)** |  | **Negative**  **n (%)** | **Positive**  **n (%)** |  |
| **Gender** | Male | 179  (91.3) | 78  (87.6) | 0.391 | 180  (91.4) | 77  (87.5) | 0.389 | 195  (91.5) | 62  (86.1) | 0.178 | 242  (91.7) | 15  (71.4) | ***0.010*** |
|  | Female | 17  (8.7) | 11  (12.4) |  | 17  (8.6) | 11  (12.5) |  | 18  (8.5) | 10  (13.9) |  | 22  (8.3) | 6  (28.6) |  |
| **T-Stage** | T1 | 11  (5.6) | 10  (11.5) | 0.277 | 11  (5.6) | 10  (11.6) | 0.280 | 11  (5.2) | 10  (14.1) | 0.088 | 18  (6.8) | 3  (15.0) | 0.086 |
|  | T2 | 48  (24.5) | 21  (24.1) |  | 48  (24.4) | 21  (24.4) |  | 52  (24.5) | 17  (23.9) |  | 61  (23.2) | 8  (40.0) |  |
|  | T3 | 61  (31.1) | 21  (24.1) |  | 61  (31.0) | 21  (24.4) |  | 65  (30.7) | 17  (23.9) |  | 80  (30.4) | 2  (10.0) |  |
|  | T4 | 76  (38.8) | 35  (40.2) |  | 77  (39.1) | 34  (39.5) |  | 84  (39.6) | 27  (38.0) |  | 104  (39.5) | 7  (35.0) |  |
| **N-Stage** | N0 | 44  (22.4) | 23  (26.7) | 0.450 | 44  (22.3) | 23  (27.1) | 0.446 | 50  (23.6) | 17  (24.3) | 1.000 | 62  (23.7) | 5  (25.0) | 1.000 |
|  | ≥ N1 | 152  (77.6) | 63  (73.3) |  | 153  (77.7) | 62  (72.9) |  | 162  (76.4) | 53  (75.7) |  | 200  (76.3) | 15  (75.0) |  |
| **M-Stage** | M0 | 192  (99.5) | 77  (98.7) | 0.494 | 192  (99.5) | 77  (98.7) | 0.494 | 207  (99.5) | 62  (98.4) | 0.412 | 252  (99.2) | 17 (100.0) | 1.000 |
|  | M1 | 1  (0.5) | 1  (1.3) |  | 1  (0.5) | 1  (1.3) |  | 1  (0.5) | 1  (1.6) |  | 2  (0.8) | 0  (0.0) |  |
| **Smoking** | None | 7  (3.8) | 4  (6.7) | 0.472 | 7  (3.8) | 4  (6.7) | 0.472 | 8  (4.1) | 3  (6.5) | 0.440 | 9  (3.8) | 2  (22.2) | 0.056 |
|  | Any | 176  (96.2) | 56  (93.3) |  | 176  (96.2) | 56  (93.3) |  | 189  (95.9) | 43  (93.5) |  | 225  (96.2) | 7  (77.8) |  |
| **Alcohol** | None | 13  (7.2) | 6  (10.9) | 0.399 | 13  (7.2) | 6  (10.9) | 0.399 | 15  (7.7) | 4  (9.5) | 0.754 | 18  (7.9) | 1  (12.5) | 0.494 |
|  | Any | 168  (92.8) | 49  (89.1) |  | 168  (92.8) | 49  (89.1) |  | 179  (92.3) | 38  (90.5) |  | 210  (92.1) | 7  (87.5) |  |
| **HPV** | Negative | 182  (74.0) | 13  (34.2) | ***<0.001*** | 182  (74.0) | 13  (34.2) | ***<0.001*** | 195  (79.3) | 17  (44.7) | ***<0.001*** | 233  (94.7) | 30  (78.9) | 0.003 |
|  | Positive | 64  (26.0) | 26  (5.8) |  | 64  (26.0) | 26  (5.8) |  | 51  (20.7) | 21  (55.3) |  | 13  (5.3) | 8  (21.1) |  |

***Supplemental Table 5.*** Cross-tabulations between CEA expression and clinical factors at 1%, 5%, 10% and 50% CEA labeled cut-off values.

***Supplemental Table 6.*** Univariable and multivariable overall survival evaluating the prognostic value of CEA at 1%, 5%, 10% and 50% CEA labeled cut-off values.

| N=284 | **1%** | | **5%** | | **10%** | | **50%** | |
| --- | --- | --- | --- | --- | --- | --- | --- | --- |
|  | **Univariable OS** | **Multivariable OS** | **Univariable OS** | **Multivariable OS** | **Univariable OS** | **Multivariable OS** | **Univariable OS** | **Multivariable OS** |
| CEA Positive vs. Negative  p-value | HR = 0.67  (95% CI: 0.48-0.92)  ***p=0.011*** | HR = 0.87  (95% CI: 0.62-1.21)  *p*=0.394 | HR = 0.70  (95% CI: 0.51-0.97)  ***p=0.028*** | HR = 0.89  (95% CI: 0.64-1.24)  *p*=0.488 | HR = 0.72  (95% CI: 0.51-1.01)  ***p=0.049*** | HR = 0.90  (95% CI: 0.64-1.28)  *p*=0.549 | HR = 0.51  (95% CI: 0.27-0.97)  ***p=0.023*** | HR = 0.64  (95% CI: 0.34-1.22)  *p*=0.148 |
| HPV Positive vs. Negative  p-value | - | HR = 0.33  (95% CI: 0.19-0.58)  ***p<0.001*** | - | HR = 0.33  (95% CI: 0.19-0.57)  ***p<0.001*** | - | HR = 0.32  (95% CI: 0.19-0.56)  ***p<0.001*** | - | HR = 0.33  (95% CI: 0.19-0.57)  ***p<0.001*** |

***Supplemental Table 7.*** Cross-tabulations between CEA expression and immune marker expression at 1%, 5%, 10% and 50% labeled cut-off values.

|  |  | **1%** | | | **5%** | | | **10%** | | | **50%** | | |
| --- | --- | --- | --- | --- | --- | --- | --- | --- | --- | --- | --- | --- | --- |
| N=29 |  | **Negative**  **n (%)** | **Positive**  **n (%)** | **P=** | **Negative**  **n (%)** | **Positive**  **n (%)** | **P=** | **Negative**  **n (%)** | **Positive**  **n (%)** | **P=** | **Negative**  **n (%)** | **Positive**  **n (%)** | **P=** |
| **PDL1** | Negative | 6  (85.7) | 15  (68.2) | 0.635 | 6  (85.7) | 15  (68.2) | 0.635 | 6  (75.0) | 15  (71.4) | 1.000 | 13  (72.2) | 8  (72.7) | 1.000 |
|  | Positive | 1  (14.3) | 7  (31.8) |  | 1  (14.3) | 7  (31.8) |  | 2  (25.0) | 6  (28.6) |  | 5  (27.8) | 3  (27.3) |  |
| **PD1** | Negative | 4  (57.1) | 4  (18.2) | 0.068 | 4  (57.1) | 4  (18.2) | 0.068 | 4  (50.0) | 4  (19.0) | 0.164 | 6  (33.3) | 2  (18.2) | 0.671 |
|  | Positive | 3  (42.9) | 18  (81.8) |  | 3  (42.9) | 18  (81.8) |  | 4  (50.0) | 17  (81.0) |  | 12  (66.7) | 9  (81.8) |  |
| **CD8** | Negative/Poorly infiltrating | 3  (42.9) | 9  (40.9) | 1.000 | 3  (42.9) | 9  (40.9) | 1.000 | 3  (37.5) | 9  (42.9) | 1.000 | 8  (44.4) | 4  (36.4) | 0.717 |
|  | Moderately/Strongly infiltrating | 4  (57.1) | 13  (59.1) |  | 4  (57.1) | 13  (59.1) |  | 5  (62.5) | 12  (57.1) |  | 10  (55.6) | 7  (63.6) |  |
| **FoxP3** | Negative | 5  (71.4) | 15  (68.2) | 1.000 | 5  (71.4) | 15  (68.2) | 1.000 | 6  (75.0) | 14  (66.7) | 1.000 | 14  (77.8) | 6  (54.5) | 0.237 |
|  | Positive | 2  (28.6) | 7  (31.8) |  | 2  (28.6) | 7  (31.8) |  | 2  (25.0) | 7  (33.3) |  | 4  (22.2) | 5  (45.5) |  |

***Supplemental Table 8.*** Cross-tabulations between CEA expression and immune marker expression at 1%, 5%, 10% and 50% labeled cut-off values for HPV-independent cases only.

|  | N=17 | **1%** | | | **5%** | | | **10%** | | | **50%** | | |
| --- | --- | --- | --- | --- | --- | --- | --- | --- | --- | --- | --- | --- | --- |
|  |  | **Negative**  **n (%)** | **Positive**  **n (%)** | **P=** | **Negative**  **n (%)** | **Positive**  **n (%)** | **P=** | **Negative**  **n (%)** | **Positive**  **n (%)** | **P=** | **Negative**  **n (%)** | **Positive**  **n (%)** | **P=** |
| **PDL1** | Negative | 6  (85.7) | 8  (80.0) | 1.000 | 6  (85.7) | 8  (80.0) | 1.000 | 6  (85.7) | 8  (80.0) | 1.000 | 10  (83.3) | 4  (80.0) | 1.000 |
|  | Positive | 1  (14.3) | 2  (20.0) |  | 1  (14.3) | 2  (20.0) |  | 1  (14.3) | 2  (20.0) |  | 2  (16.7) | 1  (20.0) |  |
| **PD1** | Negative | 4  (57.1) | 3  (30.0) | 0.350 | 4  (57.1) | 3  (30.0) | 0.350 | 4  (57.1) | 3  (30.0) | 0.350 | 6  (50.0) | 1  (20.0) | 0.338 |
|  | Positive | 3  (42.9) | 7  (70.0) |  | 3  (42.9) | 7  (70.0) |  | 3  (42.9) | 7  (70.0) |  | 6  (50.0) | 4  (80.0) |  |
| **CD8** | Negative/Poorly infiltrating | 3  (42.9) | 7  (70.0) | 0.350 | 3  (42.9) | 7  (70.0) | 0.350 | 3  (42.9) | 7  (70.0) | 0.350 | 7  (58.3) | 3  (60.0) | 1.000 |
|  | Moderately/Strongly infiltrating | 4  (57.1) | 3  (30.0) |  | 4  (57.1) | 3  (30.0) |  | 4  (57.1) | 3  (30.0) |  | 5  (41.7) | 2  (40.0) |  |
| **FoxP3** | Negative | 5  (71.4) | 7  (70.0) | 1.000 | 5  (71.4) | 7  (70.0) | 1.000 | 5  (71.4) | 7  (70.0) | 1.000 | 9  (75.0) | 3  (60.0) | 0.600 |
|  | Positive | 2  (28.6) | 3  (30.0) |  | 2  (28.6) | 3  (30.0) |  | 2  (28.6) | 3  (30.0) |  | 3  (25.0) | 2  (40.0) |  |
