# Supplementary material for: Exploring targets in oropharyngeal cancer – association with immune markers and AI‐scoring of B7‐H3 expression
Source: Clin Transl Med. 2025 Mar 12;15(3):e70265. doi: 10.1002/ctm2.70265 (PMC11897723; doi:10.1002/ctm2.70265)
Supplement: Supplementary file 3 — Supporting Information [file CTM2-15-e70265-s001.docx]

**Supplemental Information**

**Supplemental Methods**

*Patient Cohort*

Formalin fixed paraffin embedded (FFPE) tumor specimens were collected from OPSCC patients diagnosed between 1990 and 2016 from four independent cohorts: University College London (UCL, UK), Hospital Universitario Central de Asturias (HUCA, Spain), University Hospital Giessen (UHG, Germany) and Medical University of Innsbruck (MUI, Austria). All patients were managed per their corresponding institutional standard. Local ethical approval was obtained for each institution: UCL/UCLH (UCL/UCLH Ethics Committee, 04/0099), HUCA (Ethical Committee of HUCA, 141/19), UHG (Ethics Committee of Giessen, AZ 95/15) and MUI (AN2014-0241, 340/4.2); multi-institutional analysis was performed in line with multicenter ethics obtained from UCL (UCL REC no. 9609/002).

Surgical tissue specimen comprised archival FFPE tumor specimen obtained retrospectively from patients who had undergone surgery or biopsy for oropharyngeal squamous cell carcinoma. All specimens were treatment-naïve. At UCL and MUI, specimens were assessed as single whole slides; tissue microarrays (TMAs) were generated for HUCA and UHG. Three and four morphologically representative areas were used for TMA construction for HUCA and UHG, respectively. Staining protocols were the same across all centers.

Corresponding de-identified clinical data, including patient demographics, were obtained by report review. HPV status was evaluated previously using p16 immunohistochemistry and group-specific Alpha-HPV-PCR with hybridization-based HPV-typing.^30^ Specimens were considered HPV positive if they showed positivity on both p16 and PCR testing.

*Multiplex immunohistochemical staining*

Multiplex immunohistochemistry was performed on the Leica BOND Rx^m^. Antibodies used were anti-PDL1 (Cell Signalling Technology, no. 13684), anti-CD8 (Novocastra, no. PA0183), anti-PD1 (Cell Marque, no. 315M) and anti-FoxP3 (eBioscience, no. 14-4776-82). Briefly, tissue sections were incubated in BOND Epitope Retrieval Solution 2 for 30 minutes and stained with anti-PDL1 using the BOND Polymer Refine Detection kit with 15 minutes of antibody incubation. This was followed anti-CD8 incubation for 15 minutes using the BOND Polymer Refine Red Detection kit. Anti-FoxP3 was subsequently applied to the tissue, again for 15 minutes using the BOND Polymer Refine Red Detection kit however the green chromogen was developed using HIGHDEF green IHC Chromogen (AP) from Enzo. Lastly, the tissue was incubated with anti-PD1 for 15 minutes and again developed using the HIGHDEF green IHC chromogen (AP) from Enzo.

*Immunohistochemical Staining*

Automated immunohistochemistry was performed on 3µm sections. All automated staining using anti-B7-H3 (Cell Signalling Technology, no. 14058) and anti-CEA (Cell Signaling Technology, no. 2383) antibodies were carried out on the Leica BOND Rx^m^. All staining was performed using the Leica Bond Polymer Refine detection system (Leica Biosystems, DS9800) according to manufacturer recommendations.

*Semi-quantitative scoring of IHC slides*

B7-H3 and CEA expression were separately semi-quantitatively graded by independent consultant histopathologists blinded to clinical factors and outcome. For B7-H3, intensity was graded as 0 (no expression or <10%), 1 (weak expression), 2 (moderate expression) or 3 (strong expression). Specimens were classed as B7-H3 positive if assigned an intensity score of at least 1, i.e. ≥10% of cells stained. CEA expression was evaluated by percentage of tumor cells stained. Immune markers were separately semi-quantitatively scored. PD1, PDL1 and FoxP3 were each scored as either positive or negative. The presence of CD8+ T-cells were scored as negative, poorly infiltrating, moderately infiltrating, or strongly infiltrating.

*Development of QuPath Pipeline and Digital Scoring of B7-H3 Expression*

The open-source digital pathology software QuPath was used to evaluate the extent of B7-H3 immunohistochemical staining in the extended cohort (Supplemental Figure 1).^31^ A custom script was developed in the Groovy programming language. Firstly, the image was deconvolved to digitally separate the DAB and hematoxylin stains. Regions of tissue, white space, and artefact were manually annotated on a subset of sections, which was then used to train a pixel classifier to identify tissue. The StarDist extension, a deep-learning model, was then used to detect cell nuclei and make cell level measurements.^32^ The minimum nucleus area and minimum nucleus intensity (using the mean nuclear hematoxylin intensity) were set at 5 um^2^ and 0.022, respectively. The maximum nucleus area was set at 1300 um^2^. An object classifier was then trained on manually annotated areas of tumor and stroma and integrated into the pipeline to determine the extent of DAB staining in the respective regions. The output of the analysis included the number of cell detections and H-score. The H-score is the sum of the percentage of cells stained multiplied by the degree of intensity, yielding scores within a range of 0 and 300.^33^ Thresholds for intensity were calculated using the means and standard deviations of DAB staining and applied to slides using QuPath’s intensity classification function. Given the variability between centers in slide preparation and staining, this was done separately for each center. H-score was used as the primary measure of B7-H3 staining. Where several specimens were available for a single case (i.e., TMAs), the average H-score was taken.

*Ethics Statement*

Local ethical approval was obtained for each institution: UCL/UCLH (UCL/UCLH Ethics Committee, 04/0099), HUCA (Ethical Committee of HUCA, 141/19), UHG (Ethics Committee of Giessen, AZ 95/15) and MUI (AN2014-0241, 340/4.2); multi-institutional analysis was performed in line with multicenter ethics obtained from UCL (UCL REC no. 9609/002). Informed consent was obtained from all patients.

*Statistical Analysis*

Patient and clinical characteristics are presented as summary statistics. Associations between factors were assessed using the Fisher’s Exact Test or Chi-Square Test, where appropriate. Prognostic value was evaluated in terms of overall survival (OS) and disease-free survival (DFS), calculated from the date of diagnosis, and censored at the date the patient was last known to be alive if no event had occurred. Cox regression analyses were used to derive hazard ratios, 95% confidence intervals and corresponding p-values, both unadjusted and after accounting for other factors. The Kruskal Wallis non-parametric test was used to ascertain the relationship between semi-quantitative and quantitative B7-H3 scores with test statistics, degrees of freedom and *p*-values presented. Associations between immune marker status and B7-H3 scores were similarly assessed, where relevant. The Mann Whitney-U test was used to assess associations between B7-H3 H-score and binary variables. The data analysis was generated using IBM SPSS Statistics for Windows version 27.0 (IBM Corp., Armonk, NY, USA).

**Supplemental Results**

The prognostic value of various clinical factors largely aligned with previous reports and are presented in Supplemental Table 1. Both T-stage and HPV-status were significantly prognostic and, strikingly, on stratification of the former by the latter, a similar overall survival outcome was observed between HPV-positive T4 cases and HPV-negative T1 disease (Supplemental Figure 3).

Tissue from an initial cohort comprising thirty-four cases were comprehensively assessed for immune microenvironment markers (Supplemental Table 2a). Tumoral PDL1 and PD1 expression was observed in 23.5% (8/34) and 70.6% (24/34) of samples, respectively. 2.9% (1/34) of samples were negative for infiltrating CD8+ T-cells; 38.2% (13/34) were poorly infiltrating, 41.2% (14/34) were moderately infiltrating and 17.6% (6/34) were strongly infiltrating. 35.3% (12/34) of samples were positive FoxP3+ T-cells. On univariable survival analysis, only increasing infiltration of CD8+ T-Cells (HR = 0.41, 95% CI: 0.18-0.95, *p* = 0.040) and the presence of FoxP3+ T-cells (HR = 0.34, 95% CI: 0.11-0.99, *p* = 0.028) were associated with improved overall survival (Supplemental Table 2b). Multivariable survival analyses to account for HPV-status as a potential confounding variable was not performed due to the small sample size. Nevertheless, both PD1 tumoral expression and increasing CD8+ T-cell infiltration were significantly associated with positive HPV-status (Supplemental Table 2c). On multiplex immunohistochemistry, high expression of PDL1 in tumor cells was observed in the HPV-positive samples, alongside a rich population of CD8+ T-cells and an increase in the presence of FoxP3-expressing T-regulatory cells. Conversely, the HPV-negative samples demonstrated very low expression of PD-L1 with tumor-associated CD8+ T-cells present and occasional T-regulatory cells (Supplemental Figure 2).

PD1, PDL1, CD8, FoxP3 expression was observed (Supplemental Table 2a). Tumoral PDL1 and PD1 expression was observed in 23.5% (8/34) and 70.6% (24/34) of samples, respectively. 2.9% (1/34) of samples were negative for infiltrating CD8+ T-cells; 38.2% (13/34) were poorly infiltrating, 41.2% (14/34) were moderately infiltrating and 17.6% (6/34) were strongly infiltrating. 35.3% (12/34) of samples were positive FoxP3+ T-cells. On univariable survival analysis, only increasing infiltration of CD8+ T-Cells (HR = 0.41, 95% CI: 0.18-0.95, p = 0.040) and the presence of FoxP3+ T-cells (HR = 0.34, 95% CI: 0.11-0.99, p = 0.028) were associated with improved overall survival (Supplemental Table 2b). Both PD1 tumoral expression and increasing CD8+ T-cell infiltration were significantly associated with positive HPV-status (Supplemental Table 2c).

B7-H3 H-scores were cross-tabulated with clinical factors, HPV status and immune marker expression (Supplemental Table 3). On univariable analysis, tumoral and stromal B7-H3 H-scores significantly correlated with gender, smoking and alcohol history, T-stage, grade, and HPV-status. Furthermore, cross-tabulation of B7-H3 was done in relation to clinical factors and immune markers in HPV-independent cases. When evaluating HPV-independent cases only, any alcohol history, M0-stage and lower tumor grade (i.e. more well-differentiated tumors) were each associated with higher B7-H3 tumor expression. Higher stromal B7-H3 scores were also observed with lower tumor grade (Supplemental Table 4).

CEA expression was also cross-tabulated with clinical factors at cut-off values. Several labelled cut-off values (1%, 5%, 10% and 50%) were explored to evaluate associations between CEA across the tissue section and other clinical factors (i.e. gender, T-stage, N-stage, M-stage, smoking/alcohol history, HPV status and overall survival). No significant correlations were observed except between CEA expression and HPV-status, which were significantly associated at all cut-off values, as well as between CEA expression and gender at the 50% cut-off value (Supplemental Table 5). On univariable analysis, CEA at each cut-off was significantly predictive of overall survival, with positive expression correlating with superior survival. However, this significance was lost after adjusting for HPV status (Supplemental Table 6). Moreover, CEA did not significantly correlate with any of PD-1, PDL1, CD8+ T-cell infiltration nor FoxP3 expression (Supplemental Table 7) .
